# Spinal Intradural-Extramedullary Neurocysticercosis: A Case Report

**DOI:** 10.7759/cureus.84397

**Published:** 2025-05-19

**Authors:** João M Fonseca, Lucca R Borba, Ana S Chiaretti, Marilia C Resende, Luciano E Fonseca Junior

**Affiliations:** 1 Radiology, University of Florida, Gainesville, USA; 2 Internal Medicine, Hospital Universitário Professor Edgard Santos, Salvador, BRA; 3 Internal Medicine, Federal University of Bahia, Salvador, BRA; 4 Family Medicine, Federal University of Bahia, Salvador, BRA; 5 Pathology, Hospital Mater Dei, Salvador, BRA

**Keywords:** extraparenchymal neurocysticercosis, infectious and parasitic diseases, intradural-extramedullary tumor, spinal cysticercosis, spinal mri

## Abstract

Neurocysticercosis is a parasitic infection of the central nervous system (CNS) caused by the larval stage of the *Taenia solium* tapeworm. This condition is most commonly characterized by the development of cysts in the intracranial CNS, causing a wide range of neurological symptoms, such as seizures, headaches, and signs of increased intracranial pressure. Spinal intradural-extramedullary neurocysticercosis is a rare extracranial form of the disease that often resembles other conditions, such as arachnoid cysts or spinal tumors. We report the case of a 55-year-old man from Bahia, Brazil, who experienced chronic urinary retention, lower back pain, and bilateral leg tingling. Original MRI scans suggested multiple arachnoid cysts, but further imaging indicated neurocysticercosis as a possible diagnosis. The patient underwent T12-S1 laminectomy, where cystic lesions were identified and biopsied. Histopathology, posteriorly, confirmed the final diagnosis of neurocysticercosis, showing areas of necrosis and calcifications and viable parasite structures. After surgery, the patient still presented mild symptoms, such as constipation and left leg paresthesia. This case highlights the challenges of spinal neurocysticercosis diagnosis. We emphasize the importance of considering it in the differential diagnosis of spinal cystic lesions, especially in areas where the disease is common.

## Introduction

Background

Cysticercosis is the most common central nervous system (CNS) helminthic infection. It is caused by the ingestion of *Taenia solium* eggs via fecal-oral transmission [1]. It is endemic to underdeveloped regions such as Asia, Sub-Saharan Africa, and Latin America. Still, its incidence in developed areas such as North America and Europe has increased, mainly due to rising immigration from endemic locations [1,2].

Clinical and radiological presentations

There is a wide variety of clinical and radiologic presentations, with headache and seizures as the most common symptoms in the parenchymal variation of the disease [1]. Extra-parenchymal disease, which occurs outside the brain tissue, generally has a poorer prognosis and is seen in a smaller percentage of cases (9% of all neurocysticercosis cases), with symptoms dependent on the location of cyst deposition: ventricular (6%), subarachnoid, and spinal cysts (0.2%) [3,4]. CT offers high specificity and sensitivity, especially when identifying the calcified nodular stage of disease, while the MRI displays higher contrast resolution and increased strength for detection [3]. There are five disease stages ranging from a non-cystic, radiologically occult, initial lesion, moving towards the calcified nodular appearance, with progressive rim-enhancement and edema until loss of enhancement in the latter stage [3]. A central scolex can also be seen in nearly half of the cases [3].

Spinal cysticercosis

Spinal cysticercosis is a rare entity with a frequency ranging from 0.25% to 5.8%. It can be classified into extradural, intradural-extramedullary, and intramedullary [2]. One of the most important mechanisms for disease manifestation is the mass effect causing radiculopathy, myelopathy, or both, and the inflammatory reaction the dying larvae generate [4]. This is particularly true for the intradural-extramedullary variant of spinal cysticercosis, which presents with commonly mistaken overlapping imaging and clinical features as arachnoid cysts, intradural tumors, tuberculomas, or other parasitic infections, especially without a previous documented exposure history or parenchymal concomitant lesions [4].

Differential diagnoses for spinal cysticercosis

In addition to the parenchymal form of neurocysticercosis, several spinal-specific conditions may mimic its presentation due to overlapping clinical and imaging features. Spinal neuroschistosomiasis is more symptomatic than its cerebral form and typically presents as acute or subacute myelopathy or myeloradiculopathy, with heterogeneous gadolinium enhancement and T2 hyperintensity [5,6]. Similarly, spinal echinococcosis can present with back pain and limb weakness, with imaging findings of calcified or cystic lesions that resemble neurocysticercosis [7]. These conditions, though rare, are important to consider in the differential diagnosis of spinal neurocysticercosis.

## Case presentation

A 55-year-old male, born in Vereda and residing in Irarós (Bahia, Brazil), without a history of chronic diseases and with a past medical history of urinary retention, weak urinary stream, and post-void dribbling since 2020, two years before the present admission, which was attributed to benign prostatic hyperplasia. The patient underwent a prostatectomy at the time, which showed slight improvement, followed by an indwelling urinary catheter and, later, a cystectomy (surgical removal of the bladder). He also noticed the onset of constipation along with the urinary symptoms. Shortly after the surgery, he reported lower back pain that radiated to the lower limbs, associated with tingling and bilateral paresthesia that responded partially to first-line anesthetic agents. There were no complaints of lower limb weakness or diminished sensitivity. A review of systems was positive for a 10 kg (22 lbs) weight loss without any other systemic complaints, such as fever or fatigue. After evaluation with a neurologist, he was prescribed Gabapentin 300mg Bid and ordered an MRI.

Neurological examination was relevant for diminished strength in the lower limbs and hypoesthesia at the knee level. The calcaneal reflex was absent bilaterally, and the patellar reflex was present bilaterally.

**Figure 1:**
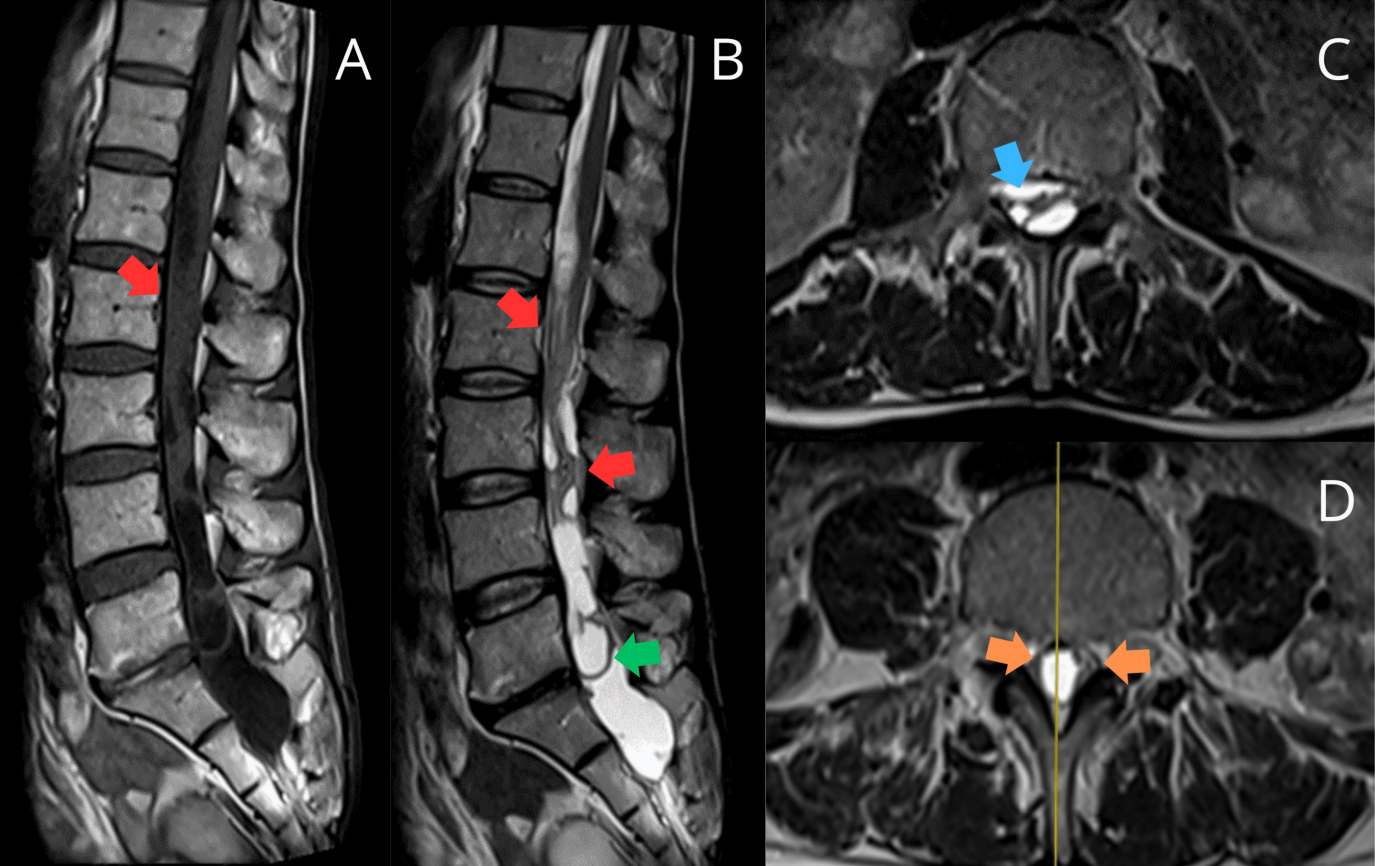
MRI shows adhesive arachnoiditis with grouped and distorted cauda equina nerve roots. Some roots are adhered to the dural sac (red arrows), while others are in the subarachnoid space. Loculations (green arrow) are present as well as central (blue arrow) and peripheral (orange arrows) nerve grouping. No racemose cyst with scolex is observed. Final report: adhesive arachnoiditis, likely of inflammatory-infectious origin A: T1 sagittal view, B: T2 sagittal view, C-D: T2 axial view MRI: magnetic resonance imaging

He underwent an MRI of the lumbar spine in June 2021 that showed multiple, poorly defined, intradural-extramedullary cystic formations of lobulated aspect with thick septations and wall enhancement that occupied the totality of the medullary canal and extended from T12 to S2. The medulla and nerve roots were compressed from the cauda equina and vertebral canal widening in the L5-S2 segment. These findings strongly suggested multiple arachnoid cysts that could be idiopathic or the result of a past infectious process (Figure 1). On the subsequent evaluation of the performed MRI, a diagnosis of adhesive arachnoiditis was established, considering the distortion and grouping of the cauda equina's nerve roots, with adhesions to the dural sac and subarachnoidal nerve root grouping, displaying loculations along the subarachnoid space.

Spinal imaging was redone after six months, in January 2022, and the findings were concordant with the prior exam. This raised the suspicion of infectious adhesive arachnoiditis secondary to intradural neurocysticercosis. The neurosurgery and neurology teams evaluated the patient to determine the appropriate treatment.

In November 2022, the patient underwent a T12-S1 laminectomy. During the procedure, it was noted that the lesion resembled neurocysticercosis cysts and extended to the exterior of the Dura mater at the sacral level. The cysts were biopsied and sent to the pathology laboratory for further evaluation. The surgery was performed without complications, and the patient was discharged two days later.

In the macroscopic evaluation, several irregular white tissue fragments measured a combined 4.8 x 4.5 x 1.0 cm from an intradural-extramedullary lesion. There was also an irregular fragment of brown fibroelastic tissue measuring 1.2 x 0.5 x 0.5 cm from the sacral lesion.

**Figure 2:**
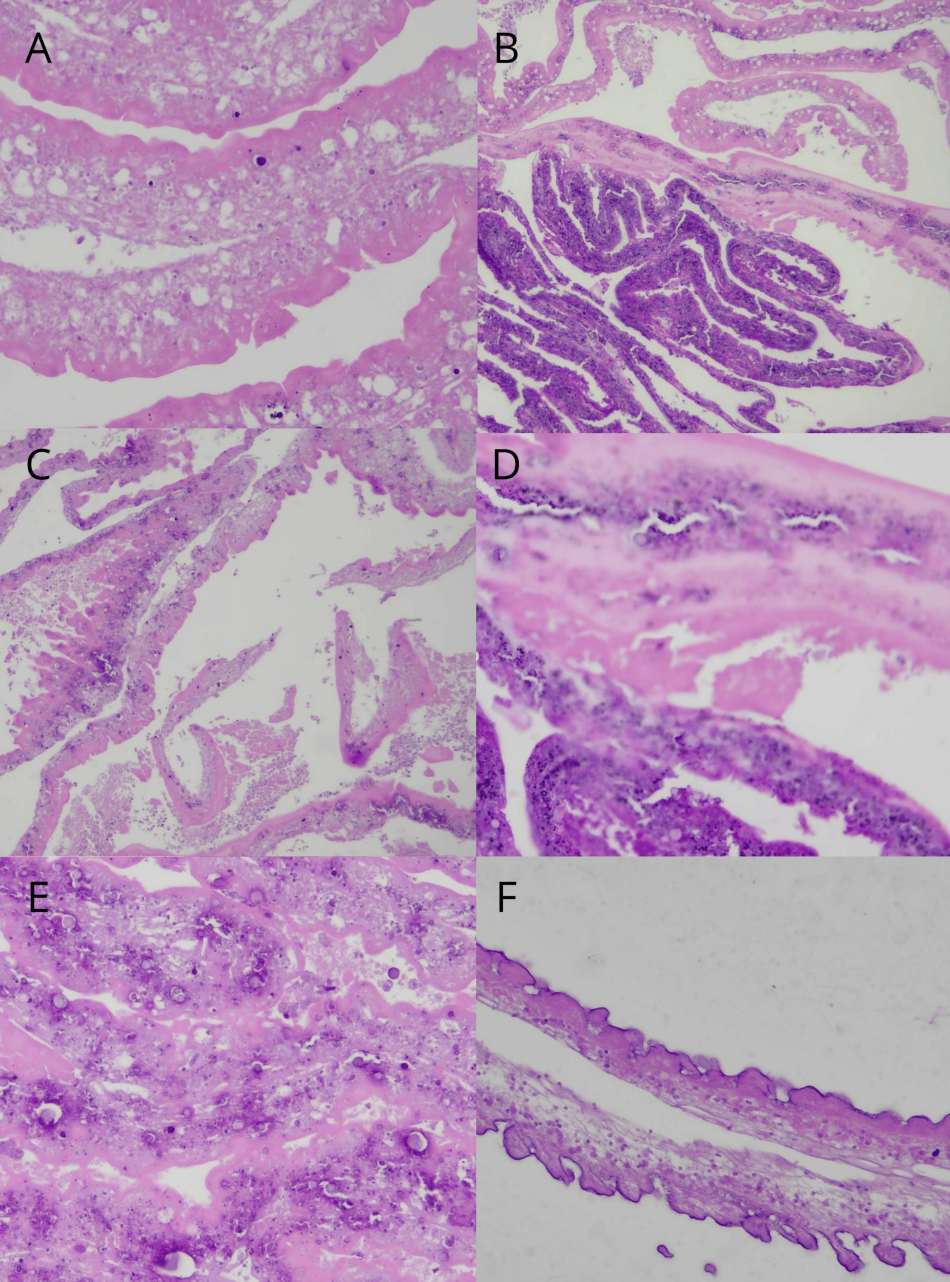
Histopathological examination of the intradural and sacral lesions shows necrosis, calcification, and viable parasite structures with circumvolutions of hyaline material and microvilli-like projections. Reactive gliosis is noted. The sacral lesion displays chronic inflammation in the dura mater and vascular neoformation A, C: H&E at 20x original magnification, B: H&E at 10x original magnification, D-F: H&E at 40x original magnification H&: hematoxylin and eosin

The microscopic evaluation of the intradural lesion evidenced areas of coagulative necrosis (Figure 2A-2D) alternating with calcification (Figure 2E) and other viable areas. On further evaluation, we identified the tegumental wall of the parasitic structure, which exhibits multiple circumvolutions and is constituted of hyaline homogenous material. In other places, the internal surface of the circumvolutions exhibited inward projections characterized as microvilli (Figure 2F). Reactive gliosis was noted. The analysis of the sacral lesion showed dura mater, chronic inflammation, and vascular neoformation. Based on microscopic characteristics, the diagnosis of cysticercosis and unspecified chronic meningitis was made.

​​​​​​At the postoperative appointment, the patient reported persistency of constipation, left lower limb paresthesia, and pain, as well as a new-onset burning sensation and tingling noted on the right lower limb.

## Discussion

Challenges in diagnosis

Spinal neurocysticercosis, notably isolated intradural-extramedullary or intramedullary, is the rarest form of cysticercosis, accounting for 0.7-5.85% of all cases reported. Estimates on prevalence are limited due to the disease being endemic in low- and middle-income countries, but the World Health Organization estimates that neurocysticercosis is responsible for 30% of epilepsy cases in those countries (totaling 2.5-8.3 million cases worldwide). The symptoms range from vague pain, radiculopathy, paresthesia, numbness, and urinary frequency to frank cauda equina syndrome, as observed in different case reports [8-10].

As the clinical symptoms are highly variable and nonspecific, the absolute diagnosis of spinal neurocysticercosis comes from visual histological confirmation of the parasite, which is rarely possible due to the invasiveness of the procedure required to access the cysts [11]. Current diagnostic strategies rely on imaging studies and serologic testing. MRI provides the best image definition to identify cyst number, size, location, and surgical planning, with the common finding of hypointense T1 and hyperintense T2-weighted images [8-11]. It is a significant advance from the already revolutionary CT that allowed visualization of the structures in the 1970s, mainly to observe brain calcifications [11].

Spinal adhesive arachnoiditis, although rare, is another critical differential imaging pattern. It has multiple possible etiologies, mainly CNS infections, traumatic lesions, spinal surgery, autoimmune disorders (such as ankylosing spondylitis), and pharmacologic exposure. Nowadays, spinal surgery is the leading cause of adhesive arachnoiditis, located predominantly in the lumbar region. Even though most cases are asymptomatic, the clinical presentation is broad, including paresthesia, lower limb weakness, diminished reflexes, lumbar or radiating pain, and autonomic disabilities (such as sphincter dysfunction), as seen in our case. Diagnosis requires thorough history assembly and clinical examination alongside contrasted MRI, which may show pseudocysts with adherent and narrow nerve roots toward the center of the dural sac or peripherally clustered and narrow nerve roots with an empty thecal sac; the cysts may also be uniloculated or multiloculated, similar to our patient. There is no established treatment for this ailment, and it most commonly consists of anti-inflammatory agents, such as nonsteroidal anti-inflammatory drugs and pulse corticosteroid therapy. Surgical approaches have been proposed, but none have shown superiority [12].

Immunology also plays a key role in diagnosis, with the lentil lectin glycoprotein enzyme-linked immunoelectrotransfer blot performing at a sensitivity of 98% and specificity of 100% as early as five weeks post-infection. However, it is limited in availability and lower in sensitivity in the presence of a single cyst. Serum and CSF enzyme-linked immunosorbent assay (ELISA) antigen detection is a more viable option. Still, it is less sensitive, notably with a lower disease burden, and is used more for treatment monitoring [11].

For the past 25 years, the diagnosis of cysticercosis has followed a revised algorithm based on absolute, major, minor, and epidemiologic factors to stratify diagnostic certainty as definitive, probable, and possible [11]. A definitive diagnosis requires either an absolute criterion, such as histological or subretinal visualization of the parasite or demonstration of a cystic lesion with a central scolex; two primary neuroimaging criteria, such as a cystic or enhancing lesion plus an epidemiologic/clinical factor; one major and one confirmative neuroimaging criterion, such as the resolution of a cystic lesion post therapy or spontaneous resolution of a small enhanced lesion plus an epidemiologic/clinical factor; or one major neuroimaging criterion plus two epidemiologic/clinical factors, such as positive serology and location in an endemic area plus exclusion of other similar pathologies [11].

Differential diagnosis

Differentiating similar pathologies can be challenging, as other parasitic infections might cause cystic lesions on the spine, either on the vertebrae, adjacent structures, or inside the medullary canal. Furthermore, these parasites are endemic to the same regions of the globe as taeniasis, adding complexity to the clinical distinction (Table 1).

**Table 1:** Imaging and histological features of differential diagnoses for spinal neurocysticercosis

Disease	Imaging	Histology
Neurocysticercosis	Multiple cysts that are hypointense on T1 and hyperintense on T2-weighted images	On macroscopic examination, cysts are white and grape-sized. Microscopically, cysts have a fibrous capsule with the parasite’s scolex inside. Calcification might be seen
Schistosomiasis	Signs of edema and heterogeneous contrast enhancement	Small, white, pinhead-sized cysts. On microscopy, intense fibrosis surrounding an egg might degenerate and calcify
Echinococcosis	Solitary, well-circumscribed, multiloculated, fluid-filled masses, usually with a homogeneous external capsule. Lesions might have a double layer of calcification on CT imaging	Cysts are usually large and white-colored. On microscopic examination, cysts are surrounded by a fibrous capsule, which might be calcified, with smaller cysts inside the main lesion
Adhesive arachnoiditis	Pseudocysts with adherent and narrow nerve roots toward the center of the dural sac OR peripherally clustered and narrowed nerve roots with an empty thecal sac. Cysts might be uni- or multiloculated.	On pathologic examination, fibrosis of the dura is common. Neural tissue edema and diminished CSF flow might be present

Echinococcosis, or hydatid disease, is a zoonotic disease caused by the cestodes of *Echinococcus granulosus* and most commonly occurs in rural areas where canines and cattle co-exist [7]. The cysts are found mainly in the liver or lungs, but any body tissue may be affected. Spinal hydatid disease is a rare presentation of echinococcosis, representing 0.5-1% of the cases. The most common sites are the vertebrae or adjacent structures seeded by the porto-vertebral venous shunt. Signs and symptoms are directly related to cyst location, varying from paresthesia and movement abnormalities to paralysis. There are cases of intradural-extramedullary lesions reported in the literature, with similar presentations to ours [13-15]. Although CT and MRI can diagnose echinococcosis lesions, MRI is preferred for its greater sensitivity in identifying smaller lesions, especially after surgical intervention [7]. In imaging studies, the cysts appear as solitary, well-circumscribed, fluid-filled masses, and even though the cysts tend to be homogenous, multiloculated lesions have been described [14]. ELISA or Western blot analysis of the cystic fluid is necessary for the definitive diagnosis [7]. The treatment of choice for spinal echinococcosis is surgical resection of the cyst and systemic therapy with albendazole to prevent recurrence [7].

Schistosomiasis is a parasitic infection caused by *Schistosoma* spp. that is also endemic to Latin America. Spinal schistosomiasis is a rare presentation of this disease, primarily caused by *Schistosoma mansoni* infection. Cases of spinal involvement and transverse myelitis associated with *Schistosoma* infection have been reported both in endemic areas and in non-endemic countries in patients with travel history [6,16]. Spinal injury is more likely to occur early in the disease course, and the most common complaint is lumbar pain and lower limb paresthesia, followed by urinary retention, which was also found in our patient [17]. Diagnosis of neuroschistosomiasis (either spinal or cerebral) is only confirmed by biopsy and histological evaluation of the lesions showing ova and granulomas, and patients with neurological symptoms and positive stool or serologic tests are characterized as having “probable” neuroschistosomiasis. Imaging studies help identify brain granulomas, and spinal MRI may show signs of edema and heterogeneous contrast enhancement [17]. Treatment consists of anti-helminthic therapy with praziquantel to terminate the adult form of the parasite and corticosteroids to reduce inflammation and granuloma formation. Surgery may be necessary for those patients with signs of intracranial hypertension or those with clinical deterioration despite conservative treatment [17].

Treatment of spinal neurocysticercosis

Spinal cysticercosis treatment consists of a pharmacologic approach with albendazole (usually preferred over praziquantel due to higher CSF penetration) and corticosteroids, indicated for patients with stable neurologic status, and its duration is individualized according to treatment response. Surgical treatment is warranted in the presence of severe neurological deficits refractory to medical therapy or in cases where definitive histopathological confirmation of diagnosis is needed. Despite treatment, as evidenced in our patient, there is still severe perioperative morbidity and mortality [8,9,18].

## Conclusions

This case study highlights a rare spinal intradural-extramedullary neurocysticercosis presentation, emphasizing its complex clinical, radiological, and histological features. The diagnostic process is often ambiguous due to the overlap with other intradural pathologies, underscoring the importance of maintaining a high index of suspicion, particularly in patients from endemic regions or with relevant exposure. This case also emphasizes the critical need for a multidisciplinary collaborative approach, incorporating specialists from neurology, infectious diseases, radiology, and pathology, to ensure timely and accurate diagnosis and appropriate management. Given the diagnostic challenges, early recognition and intervention are key to improving patient outcomes.
